# Molecular characterization and phylogenetic analysis of highly pathogenic H5N1 clade 2.3.4.4b virus in Bosnia and Herzegovina

**DOI:** 10.3389/fvets.2023.1255213

**Published:** 2023-10-26

**Authors:** Sejla Goletic, Adis Softic, Jasmin Omeragic, Amira Koro-Spahic, Naida Kapo, Emina Sabic, Dragan Kasagic, Teufik Goletic

**Affiliations:** ^1^Veterinary Faculty, University of Sarajevo, Sarajevo, Bosnia and Herzegovina; ^2^PI Veterinary Institute of the Republic of Srpska “Dr Vaso Butozan”, Banja Luka, Bosnia and Herzegovina

**Keywords:** influenza a virus H5N1 subtype, 2.3.4.4b clade, Bosnia and Herzegovina, mute swan, whole genome sequencing

## Abstract

Influenza A virus continues to represent a growing problem affecting mainly birds but with an increasing number of mammal transmission events reported each year. Nevertheless, molecular characterization and phylogenetic analysis of influenza A viruses originating from all confirmed cases have not been systematically performed in all parts of the world. In this study, we investigated a sample originating from a mute swan that died in November 2021 in the northern part of Bosnia and Herzegovina with RT-qPCR and whole genome sequencing using ONT MinION. It was diagnosed as a highly pathogenic Influenza A virus, subtype H5N1 of 2.3.4.4b clade, and phylogenetic analysis revealed high sequence homology with other European HPAI H5N1 sequences at the time. The notable detected mutations in HA (N110S and T139P) and NA genes (H155Y), that facilitate the host specificity shift and enable the resistance to some antiviral drugs respectively, underscore the necessity of virus evolution surveillance. Therefore, the rapid dissemination of information, including virological and molecular data, is essential for the introduction of tailored prevention measures for infected animals, providing clearer insight and better awareness of a potential public health threat.

## 1. Introduction

Highly pathogenic influenza A virus, the causative agent of avian influenza, continues to be an ever-growing problem for both animal and human health, especially the poultry sector, with each year seeing an increased number of cases in comparison to the last. According to the Centers for Disease Control and Prevention (CDC), the number of reported outbreaks in the 2020–2021 season alone is larger than the previous 4 years combined globally (1). The number of reported outbreaks in the 2021–2022 season proved to be even higher, with 2,398 outbreaks in poultry, 2,733 outbreaks in wild birds and over 46 million birds culled due to contact with infected and/or deceased ones (2). These are the reported cases, and the real number is considered to be much higher, especially in wild birds. All the outbreaks were attributed to highly pathogenic avian influenza (HPAI) H5Nx subtype, predominantly H5N1, of clade 2.3.4.4b, which is globally distributed and can cause disease with mortality up to 100% in chickens. The potential of the virus to spill back into wild birds, often migrating waterfowl, as well as the possibility of reassortment between different virus strains which may create new, more successful and antigenically diverse strains, emphasizes the need for constant monitoring, timely reporting of results and sharing of the complete virus genome sequence (2, 3).

In Bosnia and Herzegovina (BiH), several outbreaks of the HPAI influenza virus were recorded: a 2006 outbreak of HPAI H5N1 (subclade 2.2.2) in mute swan (*Cygnus olor*), a 2017 outbreak of HPAI H5N8 (subclade 2.3.4.4b) in non-commercial backyard poultry and feral birds, and 2021 outbreak of HPAI HPN1 (subclade 2.3.4.4b) in mute swan (*Cygnus olor*) (4). After the outbreak in 2006, a national surveillance program was established to detect and report pathogenic influenza A strains that pose a threat to public health and the commercial poultry industry. However, the two reported outbreaks (2006 and 2017) resulted in the death of a large number of birds, and many more birds that had to be euthanized with CO_2_ due to direct contact with the diseased or dead ones. Thanks to the quick action and mitigation measures taken at the time, the outbreaks were localized and relatively isolated. Furthermore, despite the various outbreaks, so far there has been only one publicly available avian Influenza A virus sequence from BiH deposited in the Global Initiative on Sharing All Influenza Data (GISAID) database in 2008. Whole genome sequencing enables the analysis and genomic characterization of the complete Influenza A virus genome, thus giving important information about the circulating clade and any mutations that might change the dynamic of infection. In this study, we used RT-qPCR and whole genome sequencing to analyze both the genetic composition of a sample from the HPAI H5N1 outbreak in 2021 as well as its phylogenetic relationships with the other HPAI H5N1 at the time.

## 2. Materials and methods

### 2.1. BiH surveillance program for influenza a virus and 2021 case detection

BiH adopted and implemented procedures for surveillance of avian influenza in wild birds following Annex II of the Commission Decision 2010/367/EU (5). The objective of this surveillance is the timely detection of HPAI of the subtype H5N1 in wild birds, targeting mostly water migratory birds, to protect commercially raised poultry and safeguard veterinary public health. Geographically, surveillance is focused on the areas near the waterways, lakes and sea, areas with a high density of poultry holdings, and specifically areas where dead birds were observed.

In late October of 2021, the dead swan was found in Gradiška (45.14737261, 17.25519481) and submitted to the PI Veterinary Institute of the Republic of Srpska “Dr Vaso Butozan.” The tested tissue samples were positive for the M gene of the Avian Influenza A virus, after which tissue samples (brain and lung) were submitted to the NRL for Avian Influenza and Newcastle Disease at the University of Sarajevo—Veterinary faculty for further RT-qPCR analysis (confirmation, subtyping and pathotyping).

### 2.2. Nucleic acids extraction

The extraction of total nucleic acids from tissue samples was performed using the DNeasy Blood and Tissue kit (Qiagen, Hilden, Germany). The proteinase K treatment lasted 1 h on a shaking thermomixer at 56°C until the tissue was completely lysed. The rest of the extraction was performed according to the manufacturer’s instructions. Furthermore, the remaining tissue was swabbed thoroughly with cotton swabs, and the nucleic acid extraction from these swabs was performed as well, using the QIAamp Viral RNA Mini kit (Qiagen, Hilden, Germany) according to the manufacturer’s instructions. Both the extractions from tissues and from swabs were used in further analysis.

### 2.3. RT-qPCR reactions

Three rounds of RT-qPCRs were performed to confirm the type, and subsequently the subtype and pathotype of the virus. All reactions included a positive and no template control. The first RT-qPCR was a screening of the M gene of the Influenza A virus, and it was performed according to the SOP VIR 018, developed according to the work of Heine et al. (6) and published by the European Union Reference Laboratory for Avian Influenza and Newcastle Disease (IZSVe EURL AI/ND).

The second round of RT-qPCRs was employed to determine the H subtype, namely H5 and H7, required by the national legislation currently in place. Both the H5-specific RT-qPCR and H7-specific RT-qPCR were performed as described elsewhere (7, 8) and both have been published as SOPs (SOP VIR 143 and 144 respectively) by IZSVe EURL AI/ND. H5 pathotype was determined with a subsequent RT-qPCR performed as described elsewhere (9).

The third round of RT-qPCR reactions was employed to determine the N subtype, specifically the N1 and N8 subtypes, using the primers described elsewhere (10). RT-qPCR reactions for N1 and N8 subtypes were performed separately using Fast Evagreen qPCR Master Mix (Biotium, San Francisco, United States) and Power SYBR™ Green RNA-to-CT™ 1-Step Kit (Applied Biosystems, Waltham, United States). Each master mix consisted of 3.84 μL RNase-free water, 10 μL of 2x Fast Evagreen Master Mix, 1 μL of specific primer mix (final concentration of each primer: 0.5 μM), 1 μL of 1x ROX reference dye, 0.16 μL of RT Enzyme Mix (125x) and 4 μL of the extracted sample. The cycling conditions were as described elsewhere (10) with added melting point analysis at the end of cycling. The melting point consisted of one incubation step of 2 min at 95°C and subsequently increasing the temperature from 65 to 95°C, with an increment of 0.5°C/cycle. Fluorescence signals were measured and collected at the end of each extension step on the EVA channel, and continuously during the melting point analysis.

### 2.4. Whole genome sequencing

To determine the clade and perform further phylogenetic analysis, whole genome sequencing was performed using the targeted approach. This approach uses Pan-Influenza primers and Superscript III One-Step RT-PCR with Platinum Taq Reagents (Invitrogen, Waltham, United States) and has been described elsewhere (11).

The final products were quantified using the Qubit dsDNA High Sensitivity Assay (Thermo Fisher Scientific, Waltham, United States) for Qubit 4 fluorometer, and the lung tissue extraction was chosen for downstream analysis as it was the sample with the optimal concentration (≥40 ng/μL). Whole genome sequencing was performed using the Rapid Sequencing Kit SQK-RAD004 (Oxford Nanopore Technologies, Oxford, United Kingdom) of a MinION Mk1C with the same settings as the previous approach.

For sequence assembly, only the reads that passed the quality check were considered. Firstly, the primers were trimmed, and the trimmed reads were put through a pipeline with multiple Linux-based bioinformatic tools. Mapping to a reference sequence was performed with minimap2 v2.25 (r1173) (12) and a consensus sequence was assembled with samtools v1.16.1 (13) and iVar v1.3.1 (14). The obtained sequence was analyzed with the BLAST module in GISAID (15) to determine the most similar sequences. The following analysis was performed for both the HA and NA gene segments: The 50 sequences with the highest sequence identity were downloaded from GISAID and used to construct a phylogenetic tree in MEGA 11 v11.0.13 (16) using the Maximum Likelihood method (Hasegawa-Kishino-Yano substitution model, 1,000 bootstrap replicates). The IDs of all GISAID sequences used in generating the tree are available in Supplementary Tables S2, S3. We used FluSurver (17) to identify mutations in HA and NA genes that may be significant in regards to the characteristics of the virus, such as changes to the host specificity, antibody recognition sites, resistance to some antiviral drugs etc. All amino acid substitutions on HA gene have been listed in Supplementary Table S1, along with their structural interactions, reported effects (if any), occurrence and prevalence among sequences of animal and mammal origin. Finally, the whole genome sequence was uploaded to GISAID (EPI_ISL_17719670), and it will become available upon publication of this study.

## 3. Results

All samples (tissue and swab extractions) were found to be positive for M gene RT-qPCR and proceeded to HA-specific RT-qPCRs, where they were all found positive for the H5 subtype and negative for the H7 subtype. H5 pathotyping determined the samples as highly pathogenic avian influenza. NA subtyping found all samples positive for N1 and negative for N8 subtype, thus the samples were determined as a highly pathogenic influenza A virus, subtype H5N1. Furthermore, a very low Ct was observed in every positive reaction, implying a high viral load. The results from all RT-qPCR reactions are summarized in Table 1.

**Table 1:** The results of RT-qPCR analysis of several samples originating from a dead mute swan.

Gene	Ct values/Tm (°C)*			
	Brain tissue	Lung tissue	Brain tissue swab	Lung tissue swab
M gene	12.8	14.5	14.9	16.1
H5	13.2	15.4	13.8	15.9
H7	No Ct	No Ct	No Ct	No Ct
H5 HP pathotype	13.8	16.2	14.4	16.3
H5 LP pathotype	No Ct	No Ct	No Ct	No Ct
N1*	8.4/81.15	10.6/81.13	10.1/81.19	12.3/81.10
N8*	No Ct/75.72	No Ct/75.64	No Ct/75.75	No Ct/75.81

*For N1 and N8, melting temperature (Tm) results were also recorded.

The output of the targeted approach was 360.54 Mb in 548,970 reads, 86% of which were passed reads with a mean Q score of 11.6 (Table 2). The depth of coverage seemed to be high overall with the targeted approach, while the mean coverage of the entire genome was calculated to be 7,041x. Detailed information about sequence metrics is available in Table 2. Furthermore, sequencing confirmed the presence of the KRRKR-G polybasic cleavage motif sequence in the HA protein, which is associated with HPAI viruses (18).

**Table 2:** The sequence metrics obtained through targeted sequencing.

Metrics		Targeted sequencing
Total reads		548,970
% of passed reads		86%
Q score		11.5
The mean depth of coverage	PB2	1,065.5
	PB1	355.1
	PA	114.6
	HA	82.1
	NP	14.1
	NA	575.3
	MP	1,230.1
	NS	3,542.3

**Figure 1:**
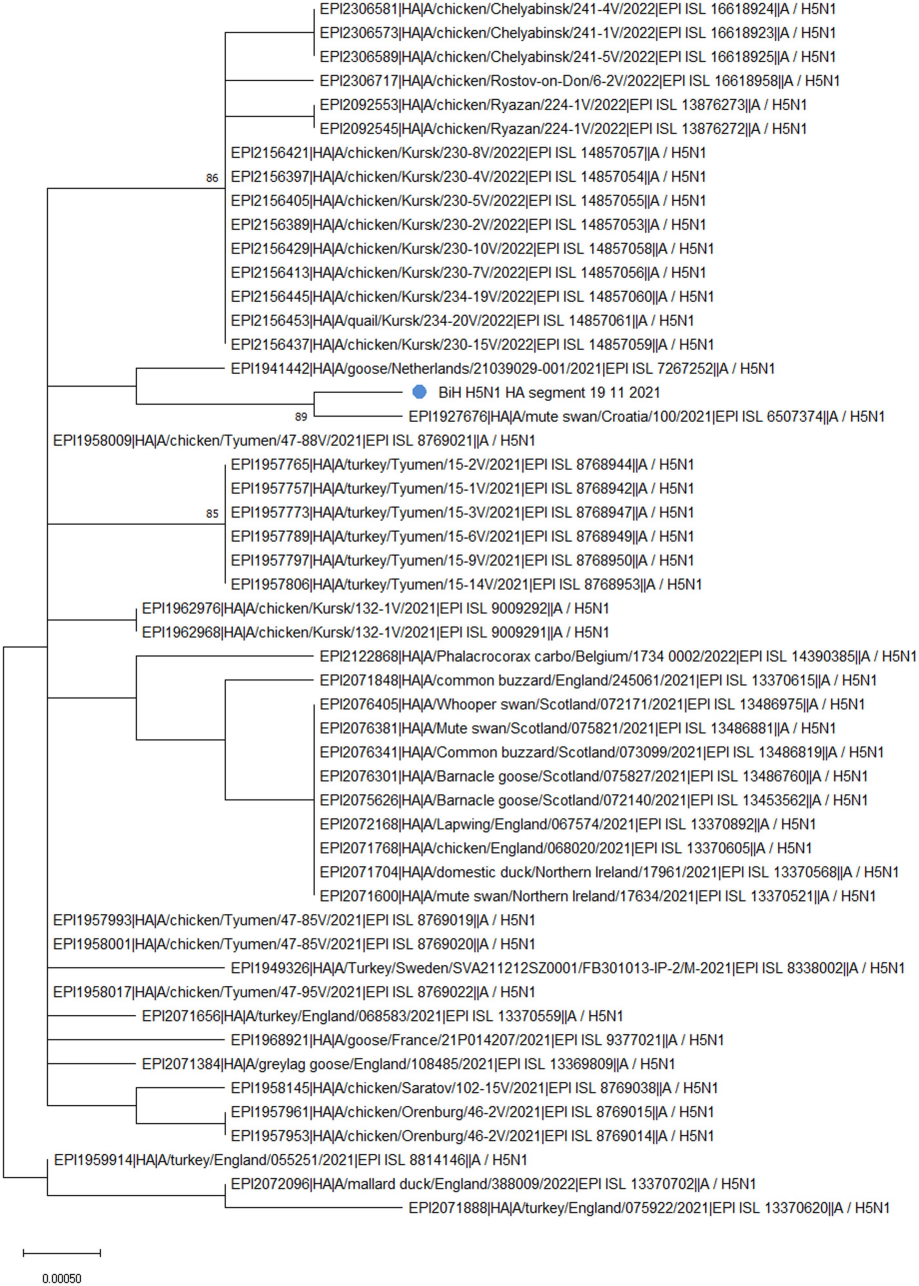
The unrooted phylogenetic tree of the HA segment of the HPAI H5N1 BiH sequence (marked with a blue dot). The tree was constructed in MEGA 11 v11.0.13 using the Maximum Likelihood method (Hasegawa-Kishino-Yano substitution model, 1000 bootstrap replicates). The percentages of replicate trees (>70%) in which the associated sequences clustered together in the bootstrap test are shown next to the branches.

Phylogenetic analysis of the HA segment revealed that the sequence from BiH (Figure 1, labeled with a blue dot) clusters with a sample from Croatia (EPI_ISL_6507374), which was also a highly pathogenic H5N1, sampled in early November 2021 from a dead mute swan. Furthermore, the sampling location in Croatia is only 30 km from the BiH border, implying that the virus circulation was established among the wild waterfowl population in the area at the time. Additionally, the phylogenetic analysis confirmed that the BiH sequence belongs to clade 2.3.4.4b and clusters with other European sequences that belong to the same clade. Interestingly, it seems that the BiH sequence forms a group with sequences originating from domestic poultry in Eastern Europe.

The analysis of the HA gene in FluSurver, using the HA sequence of A/Sichuan/26221/2014(H5N6) as a reference, revealed 18 amino acid substitutions: K3N, I10T, G16S, N110S, T139P, T156A, Q185R, V194I, A201E, N252D, E284G, M285V, I298V, D473N, K492E, V538A, I547M, and V548I. Comparison with the 50 sequences with the highest sequence identity showed the BiH HA sequence has one novel silent mutation, A1750G. However, none of the amino acid substitutions detected are novel for the BiH 2021 strain. However, D473N substitution only appears in the sequences from Bosnia, Croatia (EPI_ISL_6507374) and Netherlands (EPI_ISL_7267252), and this is reflected in the clustering of these sequences on the phylogenetic tree (Figure 1). The substitutions N110S and T139P have been previously mentioned in literature as significant since they are both in positions which make contact with cell surface glycans and thus are important for receptor binding and host specificity shift (19). A mutation in position equivalent to N110S has been reported to enable the Influenza A virus to bind to a human-type receptor more efficiently, thus facilitating host specificity shift (20). Furthermore, a mutation in position equivalent to T139P has also been reported to enable the virus to bind to a human-type receptor more efficiently (21).

**Figure 2:**
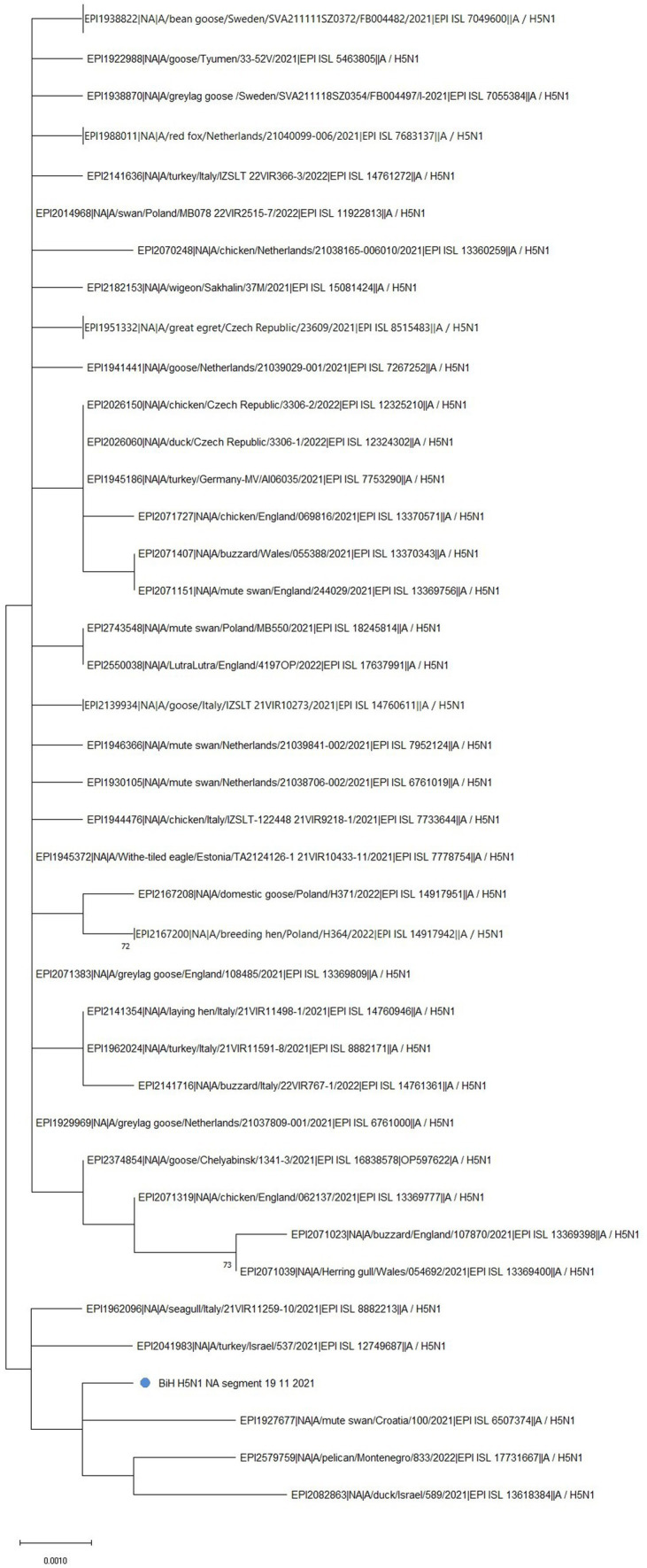
The unrooted phylogenetic tree of the NA segment of the HPAI H5N1 BiH sequence (marked with a blue dot). The tree was constructed in MEGA 11 v11.0.13 using the Maximum Likelihood method (Hasegawa-Kishino-Yano substitution model, 1000 bootstrap replicates). The percentages of replicate trees (>70%) in which the associated sequences clustered together in the bootstrap test are shown next to the branches. Some branches have been collapsed for easier viewing.

Phylogenetic analysis of the NA gene further confirmed the clustering of the BiH sequence with the Croatian one (EPI_ISL_6507374) (Figure 2, marked with a blue dot). However, the BiH sequence also forms a distinct group with NA sequences from Montenegro (EPI_ISL_17731667), Israel (EPI_ISL_13618384, EPI_ISL_12749687), and Italy (EPI_ISL_8882213) sampled in 2021/2022. This grouping seems to be determined by a point mutation T416C which is shared by these six sequences only. Furthermore, comparison with the 50 sequences with the highest sequence identity showed the BiH NA sequence has one novel silent mutation, C1437G, but no novel amino acid substitutions. In general, with the exception of the Croatian sequence, sequences that have the highest identity with BiH NA sequence completely differ than those that have the highest identity with BiH HA sequence. Having in mind the mutation rate of NA gene, this analysis implies that the reassortment of this gene happened earlier on.

In comparison to the reference NA sequence of A/Goose/Guangdong/1/1996(H5N1), the NA sequence obtained in this study contains 24 amino acid substitutions: K6R, I10T, V17I, I20V, H44Y, A46P, T76A, K78Q, A81T, V99I, H100Y, H155Y, T188I, M258I, T289M, G336S, V338M, P340S, N366S, G382E, S405T, I418M, S434N, and D460G. The mutation H155Y is especially significant, as it is associated with improved resistance to drugs such as Tamiflu and Relenza. Interestingly, it has been noted that all human N1 viruses have a conserved Y155 residue while avian N1 viruses usually have H155 (22).

## 4. Discussion

Ever since the first documented case of HPAI H5N1 in BiH in 2006 (23), an extensive annual, nationwide monitoring of the influenza A virus has been conducted both in poultry and wild bird populations, especially waterfowl and birds of prey. At the time of sampling described in this paper, there was the largest epidemic season (2021–2022) of highly pathogenic avian influenza in Europe, observed in 36 European countries, both in poultry and wild birds but also in some mammal species (2). Furthermore, the aforementioned 2021–2022 epidemic season was characterized by outbreaks of HPAI H5N1 of clade 2.3.4.4b, which is the subtype and clade of the sample described in this work. Next, HPAI virus detections in wild birds in the 2021–2022 season were predominantly in waterfowl, especially at the beginning of November when the birds migrate, but the cases in other wild birds, such as raptors, were also reported (2, 24). The HPAI H5N1 from a dead mute swan in BiH was sampled in November 2021 very close to the Croatian border. The obtained sequence shares the highest sequence homology with the sequence from Croatia, which also comes from a dead mute swan sampled in the same period. These two sequences, together with one sequence from the Netherlands, are the only ones that share the D473N substitution (Supplementary Table S2). According to the predictive computational analysis of FluSurver, this mutation might be involved in viral oligomerization interfaces and the binding of a small ligand(s). Furthermore, BiH and Croatian sequence share the H155Y substitution on the NA protein, which is associated with improved resistance to antiviral drugs. It might be important to note that both the BiH and Croatian sampling locations are separated by approximately 50 km and are both located in or closely tied to the Sava river basin, which might imply the epidemiological connection of these cases. This emphasizes the importance of sequencing and timely data sharing on available databases, such as GISAID, in responding to avian influenza outbreaks to protect both animal and human health.

The described case of HPAI H5N1 from a dead mute swan in BiH luckily did not result in a larger outbreak among other waterfowl or poultry. However, the zoonotic threat these events present cannot be ignored, especially considering the increasing number of transmission events of H5Nx viruses to mammals, including humans (2). The possibility of further adaptation of the virus to mammals and the higher occurrence of human cases make highly pathogenic influenza A virus a One Health issue, not just an economic one, and any legislation and directives regarding this virus must be revised accordingly to reflect this. In the case of BiH, it means continuous monitoring of the virus, sequencing of positive samples, updating the existing legislation as well as the preparedness/response plan to outbreaks and mitigation measures on every administrative level. In a broader international context, the findings in this study further emphasize the need for timely molecular analysis and reporting of all cases to the competent authorities and relevant international databases. Moreover, having in mind the recently reported cases of avian influenza in mammals, the implementation of the One Health approach should be addressed in the updated preparedness/response plan in BiH.

## Data availability statement

The datasets presented in this study can be found in online repositories. The names of the repository/repositories and accession number(s) can be found in the article/Supplementary material.

## Ethics statement

Ethical approval was not required for the study involving animals in accordance with the local legislation and institutional requirements because no live vertebrate/higher invertebrate animals were used in this research.

## Author contributions

SG: Conceptualization, Data curation, Formal analysis, Investigation, Methodology, Software, Validation, Visualization, Writing – original draft, Writing – review & editing. AS: Conceptualization, Data curation, Visualization, Writing – review & editing. JO: Conceptualization, Data curation, Formal analysis, Writing – review & editing. AK-S: Methodology, Visualization, Writing – review & editing. NK: Formal analysis, Investigation, Writing – review & editing. ES: Formal analysis, Investigation, Methodology, Writing – review & editing. DK: Methodology, Writing – review & editing. TG: Conceptualization, Data curation, Funding acquisition, Project administration, Resources, Supervision, Validation, Writing – review & editing.
