# Supplementary material for: Molecular characterization and phylogenetic analysis of highly pathogenic H5N1 clade 2.3.4.4b virus in Bosnia and Herzegovina
Source: Front Vet Sci. 2023 Oct 26;10:1255213. doi: 10.3389/fvets.2023.1255213 (PMC10637570; doi:10.3389/fvets.2023.1255213)
Supplement: Supplementary file 1 [file Table_1.DOCX]

Supplementary Material

**Supplementary Table S1.** Amino acid substitutions in BiH AIV sequence in comparison with the reference HA sequence of A/Sichuan/26221/2014(H5N6) and their significance.

| **Mutation** | **Alternative position according to FluSurver numbering** | **Classical H1N1 strain numbering** | **Structural interactions** | **Reported effect (reference)** | **No. of global occurrences** | **Occurrence of the mutation (%)** | **Prevalence among sequences of avian and/or mammal origin** |
| --- | --- | --- | --- | --- | --- | --- | --- |
| K3N | K4N | HA1 K13N | Viral oligomerization interfaces | N/A | 15 | 3.71 | 100% avian |
| I10T | I11T | HA1 I6T | Viral oligomerization interfaces | N/A | 2 | 0.50 | 100% avian |
| G16S | G17S | HA1 G10S | Viral oligomerization interfaces, a T-cell epitope presented by MHC molecules, binding small ligand(s), antibody recognition sites | N/A | 316 | 78.22 | 91.1% avian, 1.6% mammalian, 7.3% environment |
| N110S | N111S | HA1 N94S | Viral oligomerization interfaces, binding small ligand(s), antibody recognition sites | Host specificity shift (A mutation at the equivalent position, D94N, has been reported in Su et al., ref. 21) | 18 | 4.46 | 100% avian |
| T139P | T141P | HA1 T124P | Viral oligomerization interfaces, binding small ligand(s), antibody recognition sites | Host specificity shift (A mutation at the equivalent position, S123P, has been reported in Yamada et al., ref 22) | 346 | 85.64 | 91% avian, 1.2% mammalian, 7.8% environment |
| T156A | T158A | HA1 T141A | Viral oligomerization interfaces, binding small ligand(s), antibody recognition sites | Antigenic drift (A mutation at the equivalent position, K144E, has been reported in Van den Hoecke et al., <https://doi.org/10.1128%2FmBio.00745-21>) | 5 | 1.24 | 100% avian |
| Q185R | Q187R | HA1 Q170R | Viral oligomerization interfaces, binding small ligand(s) | N/A | 198 | 49.01 | 92.5% avian, 2.5% mammalian, 5% environment |
| V194I | V196I | HA1 V179I | N/A | N/A | 393 | 97.28 | 90.8% avian, 1.3% mammalian, 7.9% environment |
| A201E | A203E | HA1 A186E | Antibody recognition sites, viral oligomerization interfaces, binding small ligand(s) | N/A | 39 | 9.65 | 97.4% avian, 2.6% mammalian |
| N252D | N254D | HA1 N237D | Binding small ligand(s), viral oligomerization interfaces | N/A | 20 | 4.95 | 95% avian, 5% mammalian |
| E284G | E286G | HA1 E269G | Viral oligomerization interfaces, antibody recognition sites, binding small ligand(s) | N/A | 16 | 3.96 | 100% avian |
| M285V | M287V | HA1 M270V | Viral oligomerization interfaces, antibody recognition sites | N/A | 63 | 15.59 | 82.8% avian, 17.5% environment |
| I298V | I300V | HA1 I283V | Binding small ligand(s), viral oligomerization interfaces | N/A | 19 | 4.70 | 94.7% avian, 5.3% environment |
| D473N | D472N | HA2 D128N | Viral oligomerization interfaces, binding small ligand(s), a T-cell epitope presented by MHC molecules | N/A | 2 | 0.10 | 100% avian |
| K492E | K491E | HA2 K147E | Binding small ligand(s), viral oligomerization interfaces, antibody recognition sites | N/A | 401 | 99.26 | 89.3% avian, 1.5% mammalian, 9.2% environment |
| V538A | V537A | HA2 V193A | N/A | N/A | 108 | 1.82 | 100% avian |
| I547M | I546M | HA2 I202M | N/A | N/A | 26 | 6.44 | 100% avian |
| V548I | V547I | HA2 V203I | viral oligomerization interfaces | N/A | 4 | 0.99 | 100% avian |

**Supplementary Table S2.** Background information of H5N1 (HA segment) sequences used to generate the phylogenetic tree. The HA mutations listed are based on the comparison with the reference HA sequence of A/Sichuan/26221/2014(H5N6).

| **Isolate name** | **Isolate ID** | **Collection date** | **Host** | **HA mutations** | **Clade** | **Location** | **Authors** |
| --- | --- | --- | --- | --- | --- | --- | --- |
| A/chicken/Tyumen/47-95V/2021 | EPI_ISL_8769022 | 2021-10-12 | *Gallus gallus domesticus* | K3N, I10T, G16S, N110S, T139P, T156A, Q185R, V194I, A201E, N252D, E284G, M285V, I298V, K492E, V538A, I547M, V548I | 2.3.4.4b | Russian Federation / Tyumen Oblast | Goncharova, Natalia |
| A/chicken/Tyumen/47-88V/2021 | EPI_ISL_8769021 | 2021-10-12 | *Gallus gallus domesticus* | K3N, I10T, G16S, N110S, T139P, T156A, Q185R, V194I, A201E, N252D, E284G, M285V, I298V, K492E, V538A, I547M, V548I | 2.3.4.4b | Russian Federation / Tyumen Oblast | Goncharova, Natalia |
| A/chicken/Tyumen/47-85V/2021 | EPI_ISL_8769020 | 2021-10-12 | *Gallus gallus domesticus* | K3N, I10T, G16S, N110S, T139P, T156A, Q185R, V194I, A201E, N252D, E284G, M285V, I298V, K492E, V538A, I547M, V548I | 2.3.4.4b | Russian Federation / Tyumen Oblast | Goncharova, Natalia |
| A/chicken/Tyumen/47-85V/2021 | EPI_ISL_8769019 | 2021-10-12 | *Gallus gallus domesticus* | K3N, I10T, G16S, N110S, T139P, T156A, Q185R, V194I, A201E, N252D, E284G, M285V, I298V, K492E, V538A, I547M, V548I | 2.3.4.4b | Russian Federation / Tyumen Oblast | Goncharova, Natalia |
| A/turkey/England/068583/2021 | EPI_ISL_13370559 | 2021-12-09 | *Meleagris gallopavo* | K3N, I10T, G16S, N110S, T139P, T156A, Q185R, V194I, A201E, N252D, E284G, M285V, I298V, K492E, V538A, I547M, V548I | 2.3.4.4b | United Kingdom / England | Byrne, Alex |
| A/greylag_goose/England/108485/2021 | EPI_ISL_13369809 | 2021-11-29 | *Anser anser* | K3N, I10T, G16S, N110S, T139P, T156A, Q185R, V194I, A201E, N252D, E284G, M285V, I298V, K492G, V538A, I547M, V548I | 2.3.4.4b | United Kingdom / England | Byrne, Alex |
| A/chicken/Kursk/132-1V/2021 | EPI_ISL_9009292 | 2021-11-27 | *Gallus gallus domesticus* | K3N, I10T, G16S, N110S, T139P, T156A, Q185R, V194I, A201E, N252D, E284G, M285V, I298V, K492E, V538A, I547M, V548I | 2.3.4.4b | Russian Federation / Kursk Oblast | Goncharova, Natalia |
| A/chicken/Kursk/132-1V/2021 | EPI_ISL_9009291 | 2021-11-27 | *Gallus gallus domesticus* | K3N, I10T, G16S, N110S, T139P, T156A, Q185R, V194I, A201E, N252D, E284G, M285V, I298V, K492E, V538A, I547M, V548I | 2.3.4.4b | Russian Federation / Kursk Oblast | Goncharova, Natalia |
| A/turkey/England/055251/2021 | EPI_ISL_8814146 | 2021-11-06 | *Meleagris gallopavo* | K3N, I10T, G16S, N110S, T139P, T156A, Q185R, V194I, A201E, N252D, E284G, M285V, I298V, K492E, V538A, I547M, V548I | 2.3.4.4b | United Kingdom / Warwickshire | Byrne, Alex |
| A/quail/Kursk/234-20V/2022 | EPI_ISL_14857061 | 2022-07-11 | *Coturnix sp.* | K3N, I10T, G16S, N110S, T139P, T156A, Q185R, V194I, A201E, N252D, E284G, M285V, I298V, K492E, V538A, I547M, V548I | 2.3.4.4b | Russian Federation / Kursk Oblast | Goncharova, Natalia |
| A/chicken/Kursk/234-19V/2022 | EPI_ISL_14857060 | 2022-07-11 | *Gallus gallus domesticus* | K3N, I10T, G16S, N110S, T139P, T156A, Q185R, V194I, A201E, N252D, E284G, M285V, I298V, K492E, V538A, I547M, V548I | 2.3.4.4b | Russian Federation / Kursk Oblast | Goncharova, Natalia |
| A/chicken/Kursk/230-15V/2022 | EPI_ISL_14857059 | 2022-06-16 | *Gallus gallus domesticus* | K3N, I10T, G16S, N110S, T139P, T156A, Q185R, V194I, A201E, N252D, E284G, M285V, I298V, K492E, V538A, I547M, V548I | 2.3.4.4b | Russian Federation / Kursk Oblast | Goncharova, Natalia |
| A/chicken/Kursk/230-10V/2022 | EPI_ISL_14857058 | 2022-06-20 | *Gallus gallus domesticus* | K3N, I10T, G16S, N110S, T139P, T156A, Q185R, V194I, A201E, N252D, E284G, M285V, I298V, K492E, V538A, I547M, V548I | 2.3.4.4b | Russian Federation / Kursk Oblast | Goncharova, Natalia |
| A/chicken/Kursk/230-8V/2022 | EPI_ISL_14857057 | 2022-06-20 | *Gallus gallus domesticus* | K3N, I10T, G16S, N110S, T139P, T156A, Q185R, V194I, A201E, N252D, E284G, M285V, I298V, K492E, V538A, I547M, V548I | 2.3.4.4b | Russian Federation / Kursk Oblast | Goncharova, Natalia |
| A/chicken/Kursk/230-7V/2022 | EPI_ISL_14857056 | 2022-06-20 | *Gallus gallus domesticus* | K3N, I10T, G16S, N110S, T139P, T156A, Q185R, V194I, A201E, N252D, E284G, M285V, I298V, K492E, V538A, I547M, V548I | 2.3.4.4b | Russian Federation / Kursk Oblast | Goncharova, Natalia |
| A/chicken/Kursk/230-5V/2022 | EPI_ISL_14857055 | 2022-06-20 | *Gallus gallus domesticus* | K3N, I10T, G16S, N110S, T139P, T156A, Q185R, V194I, A201E, N252D, E284G, M285V, I298V, K492E, V538A, I547M, V548I | 2.3.4.4b | Russian Federation / Kursk Oblast | Goncharova, Natalia |
| A/chicken/Kursk/230-4V/2022 | EPI_ISL_14857054 | 2022-06-20 | *Gallus gallus domesticus* | K3N, I10T, G16S, N110S, T139P, T156A, Q185R, V194I, A201E, N252D, E284G, M285V, I298V, K492E, V538A, I547M, V548I | 2.3.4.4b | Russian Federation / Kursk Oblast | Goncharova, Natalia |
| A/chicken/Kursk/230-2V/2022 | EPI_ISL_14857053 | 2022-06-20 | *Gallus gallus domesticus* | K3N, I10T, G16S, N110S, T139P, T156A, Q185R, V194I, A201E, N252D, E284G, M285V, I298V, K492E, V538A, I547M, V548I | 2.3.4.4b | Russian Federation / Kursk Oblast | Goncharova, Natalia |
| A/goose/France/21P014207/2021 | EPI_ISL_9377021 | 2021-12-23 | *Anser anser* | K3N, I10T, G16S, N110S, T139P, T156A, Q185R, V194I, A201E, N252D, E284G, M285V, I298V, K492E, V538A, I547M, V548I | 2.3.4.4b | France / Midi-Pyrenees / Departement des Hautes-Pyrene | Briand, Francois-Xavier |
| A/chicken/Saratov/102-15V/2021 | EPI_ISL_8769038 | 2021-11-04 | *Gallus gallus domesticus* | K3N, I10T, G16S, N110S, T139P, T156A, Q185R, V194I, A201E, N252D, E284G, M285V, I298V, K492E, V538A, I547M, V548I | 2.3.4.4b | Russian Federation / Saratov Oblast | Goncharova, Natalia |
| A/chicken/Orenburg/46-2V/2021 | EPI_ISL_8769015 | 2021-10-15 | *Gallus gallus domesticus* | K3N, I10T, G16S, N110S, T139P, T156A, Q185R, V194I, A201E, N252D, E284G, M285V, I298V, K492E, V538A, I547M, V548I | 2.3.4.4b | Russian Federation / Orenburg Oblast | Goncharova, Natalia |
| A/chicken/Orenburg/46-2V/2021 | EPI_ISL_8769014 | 2021-10-15 | *Gallus gallus domesticus* | K3N, I10T, G16S, N110S, T139P, T156A, Q185R, V194I, A201E, N252D, E284G, M285V, I298V, K492E, V538A, I547M, V548I | 2.3.4.4b | Russian Federation / Orenburg Oblast | Goncharova, Natalia |
| A/turkey/Tyumen/15-14V/2021 | EPI_ISL_8768953 | 2021-09-21 | *Meleagris gallopavo* | K3N, I10T, G16S, N110S, T139P, T156A, Q185R, V194I, A201E, N252D, E284G, M285V, I298V, K492E, V538A, I547M, V548I | 2.3.4.4b | Russian Federation / Tyumen Oblast | Goncharova, Natalia |
| A/turkey/Tyumen/15-9V/2021 | EPI_ISL_8768950 | 2021-09-21 | *Meleagris gallopavo* | K3N, I10T, G16S, N110S, T139P, T156A, Q185R, V194I, A201E, N252D, E284G, M285V, I298V, K492E, V538A, I547M, V548I | 2.3.4.4b | Russian Federation / Tyumen Oblast | Goncharova, Natalia |
| A/turkey/Tyumen/15-6V/2021 | EPI_ISL_8768949 | 2021-09-21 | *Meleagris gallopavo* | K3N, I10T, G16S, N110S, T139P, T156A, Q185R, V194I, A201E, N252D, E284G, M285V, I298V, K492E, V538A, I547M, V548I | 2.3.4.4b | Russian Federation / Tyumen Oblast | Goncharova, Natalia |
| A/turkey/Tyumen/15-3V/2021 | EPI_ISL_8768947 | 2021-09-21 | *Meleagris gallopavo* | K3N, I10T, G16S, N110S, T139P, T156A, Q185R, V194I, A201E, N252D, E284G, M285V, I298V, K492E, V538A, I547M, V548I | 2.3.4.4b | Russian Federation / Tyumen Oblast | Goncharova, Natalia |
| A/turkey/Tyumen/15-2V/2021 | EPI_ISL_8768944 | 2021-09-21 | *Meleagris gallopavo* | K3N, I10T, G16S, N110S, T139P, T156A, Q185R, V194I, A201E, N252D, E284G, M285V, I298V, K492E, V538A, I547M, V548I | 2.3.4.4b | Russian Federation / Tyumen Oblast | Goncharova, Natalia |
| A/turkey/Tyumen/15-1V/2021 | EPI_ISL_8768942 | 2021-09-21 | *Meleagris gallopavo* | K3N, I10T, G16S, N110S, T139P, T156A, Q185R, V194I, A201E, N252D, E284G, M285V, I298V, K492E, V538A, I547M, V548I | 2.3.4.4b | Russian Federation / Tyumen Oblast | Goncharova, Natalia |
| A/Turkey/Sweden/SVA211212SZ0001/FB301013-IP-2/M-2021 | EPI_ISL_8338002 | 2021-11-12 | *Meleagris gallopavo* | K3N, I10T, G16S, N110S, T139P, T156A, Q185R, V194I, A201E, N252D, E284G, M285V, I298V, E374G, K492E, V538A, I547M, V548I | 2.3.4.4b | Sweden / Skane Lan / Skurups Kommun | Zohari, Siamak |
| A/goose/Netherlands/21039029-001/2021 | EPI_ISL_7267252 | 2021-11-17 | *Anser anser* | K3N, I10T, G16S, N110S, T139P, T156A, Q185R, V194I, A201E, N252D, E284G, M285V, I298V, D473N, K492E, V538A, I547M, V548I | 2.3.4.4b | Netherlands / Provincie Noord-Holland | Beerens, Nancy *et al.* |
| A/mute swan/Croatia/100/2021 | EPI_ISL_6507374 | 2021-11-12 | *Cygnus olor* | K3N, I10T, G16S, N110S, T139P, T156A, Q185R, V194I, A201E, N252D, E284G, M285V, I298V, D473N, K492E, Q506K, V538A, I547M, V548I | 2.3.4.4b | Croatia / Sisacko-Moslavacka Zupanija / Muzilovcica | Savić, Vladimir |
| A/chicken/Rostov-on-Don/6-2V/2022 | EPI_ISL_16618958 | 2022-08-17 | *Gallus gallus domesticus* | K3N, I10T, G16S, N110S, T139P, T156A, Q185R, V194I, A201E, N252D, E284G, M285V, I298V, K492E, V538A, I547M, V548I | 2.3.4.4b | Russian Federation / Rostov Oblast | Goncharova, Natalia |
| A/chicken/Chelyabinsk/241-5V/2022 | EPI_ISL_16618925 | 2022-07-28 | *Gallus gallus domesticus* | K3N, I10T, G16S, N110S, T139P, T156A, Q185R, V194I, A201E, N252D, E284G, M285V, I298V, K492E, V538A, I547M, V548I | 2.3.4.4b | Russian Federation / Chelyabinsk Oblast | Goncharova, Natalia |
| A/chicken/Chelyabinsk/241-4V/2022 | EPI_ISL_16618924 | 2022-07-28 | *Gallus gallus domesticus* | K3N, I10T, G16S, N110S, T139P, T156A, Q185R, V194I, A201E, N252D, E284G, M285V, I298V, K492E, V538A, I547M, V548I | 2.3.4.4b | Russian Federation / Chelyabinsk Oblast | Goncharova, Natalia |
| A/chicken/Chelyabinsk/241-1V/2022 | EPI_ISL_16618923 | 2022-07-28 | *Gallus gallus domesticus* | K3N, I10T, G16S, N110S, T139P, T156A, Q185R, V194I, A201E, N252D, E284G, M285V, I298V, K492E, V538A, I547M, V548I | 2.3.4.4b | Russian Federation / Chelyabinsk Oblast | Goncharova, Natalia |
| A/Phalacrocorax_carbo/Belgium/1734_0002/2022 | EPI_ISL_14390385 | 2022-02-02 | *Phalacrocorax carbo* | K3N, I10T, G16S, N110G, T139P, T156A, Q185R, V194I, A201E, N252D, E284G, M285V, I298V, K492E, V538A, I547M, V548I | 2.3.4.4b | Belgium / Provincie Oost-Vlaanderen / Evergem | Van Borm, Steven *et al.* |
| A/chicken/Ryazan/224-1V/2022 | EPI_ISL_13876273 | 2022-05-28 | *Gallus gallus domesticus* | K3N, I10T, G16S, N110S, T139P, T156A, Q185R, V194I, A201E, N252D, E284G, M285V, I298V, K492E, V538A, I547M, V548I | 2.3.4.4b | Russian Federation / Ryazan Oblast | Goncharova, Natalia |
| A/chicken/Ryazan/224-1V/2022 | EPI_ISL_13876272 | 2022-05-28 | *Gallus gallus domesticus* | K3N, I10T, G16S, N110S, T139P, T156A, Q185R, V194I, A201E, N252D, E284G, M285V, I298V, K492E, V538A, I547M, V548I | 2.3.4.4b | Russian Federation / Ryazan Oblast | Goncharova, Natalia |
| A/Whooper_swan/Scotland/072171/2021 | EPI_ISL_13486975 | 2021-12-11 | *Cygnus cygnus* | K3N, I10T, G16S, N110S, T139P, T156A, V164L, Q185R, V194I, A201E, N252D, E284G, M285V, I298V, K492E, V538A, I547M, V548I | 2.3.4.4b | United Kingdom / Scotland | Byrne, Alex |
| A/Mute_swan/Scotland/075821/2021 | EPI_ISL_13486881 | 2021-12-04 | *Cygnus olor* | K3N, I10T, G16S, N110S, T139P, T156A, V164L, Q185R, V194I, A201E, N252D, E284G, M285V, I298V, K492E, V538A, I547M, V548I | 2.3.4.4b | United Kingdom / Scotland | Byrne, Alex |
| A/Common_buzzard/Scotland/073099/2021 | EPI_ISL_13486819 | 2021-12-15 | *Buteo buteo* | K3N, I10T, G16S, N110S, T139P, T156A, V164L, Q185R, V194I, A201E, N252D, E284G, M285V, I298V, K492E, V538A, I547M, V548I | 2.3.4.4b | United Kingdom / Scotland | Byrne, Alex |
| A/Barnacle_goose/Scotland/075827/2021 | EPI_ISL_13486760 | 2021-12-04 | *Branta leucopsis* | K3N, I10T, G16S, N110S, T139P, T156A, V164L, Q185R, V194I, A201E, N252D, E284G, M285V, I298V, K492E, V538A, I547M, V548I | 2.3.4.4b | United Kingdom / Scotland | Byrne, Alex |
| A/Barnacle_goose/Scotland/072140/2021 | EPI_ISL_13453562 | 2021-12-13 | *Branta leucopsis* | K3N, I10T, G16S, N110S, T139P, T156A, V164L, Q185R, V194I, A201E, N252D, E284G, M285V, I298V, K492E, V538A, I547M, V548I | 2.3.4.4b | United Kingdom / Scotland | Byrne, Alex |
| A/Lapwing/England/067574/2021 | EPI_ISL_13370892 | 2021-12-03 | *Vanellinae* | K3N, I10T, G16S, N110S, T139P, T156A, V164L, Q185R, V194I, A201E, N252D, E284G, M285V, I298V, K492E, V538A, I547M, V548I | 2.3.4.4b | United Kingdom / England | Byrne, Alex |
| A/mallard_duck/England/388009/2022 | EPI_ISL_13370702 | 2022-01-27 | *Anas platyrhynchos* | K3N, I10T, G16S, N110S, T139P, T156A, Q185R, V194I, A201E, N252D, E284G, M285V, I298V, K492E, V538A, I547M, V548I | 2.3.4.4b | United Kingdom / England | Byrne, Alex |
| A/turkey/England/075922/2021 | EPI_ISL_13370620 | 2021-12-27 | *Meleagris gallopavo* | K3N, I10T, G16S, N110S, T139P, T156A, Q185R, V194I, A201E, N252D, E284G, M285V, I298V, K492E, V538A, I547M, V548I | 2.3.4.4b | United Kingdom / England | Byrne, Alex |
| A/common_buzzard/England/245061/2021 | EPI_ISL_13370615 | 2021-12-17 | *Buteo buteo* | K3N, I10T, G16S, N110S, T139P, T156A, V164L, Q185R, V194I, A201E, N252D, E284G, M285V, I298V, K492E, V538A, I547M, V548I | 2.3.4.4b | United Kingdom / England | Byrne, Alex |
| A/chicken/England/068020/2021 | EPI_ISL_13370605 | 2021-12-14 | *Gallus gallus domesticus* | K3N, I10T, G16S, N110S, T139P, T156A, V164L, Q185R, V194I, A201E, N252D, E284G, M285V, I298V, K492E, V538A, I547M, V548I | 2.3.4.4b | United Kingdom / England | Byrne, Alex |
| A/domestic_duck/Northern_Ireland/17961/2021 | EPI_ISL_13370568 | 2021-12-10 | *Anas platyrhynchos* | K3N, I10T, G16S, N110S, T139P, T156A, V164L, Q185R, V194I, A201E, N252D, E284G, M285V, I298V, K492E, V538A, I547M, V548I | 2.3.4.4b | United Kingdom / Northern Ireland | Byrne, Alex |
| A/mute_swan/Northern_Ireland/17634/2021 | EPI_ISL_13370521 | 2021-12-06 | *Cygnus olor* | K3N, I10T, G16S, N110S, T139P, T156A, V164L, Q185R, V194I, A201E, N252D, E284G, M285V, I298V, K492E, V538A, I547M, V548I | 2.3.4.4b | United Kingdom / Northern Ireland | Byrne, Alex |

**Supplementary Table S3.** Background information of H5N1 (NA segment) sequences used to generate the phylogenetic tree. The NA mutations listed are based on the comparison with the reference NA sequence of A/Goose/Guangdong/1/1996(H5N1).

| **Isolate name** | **Isolate ID** | **Collection date** | **Host** | **NA mutations** | **Clade** | **Location** | **Authors** |
| --- | --- | --- | --- | --- | --- | --- | --- |
| A/greylag_goose/England/108485/2021 | EPI_ISL_13369809 | 2021-11-29 | *Anser anser* | K6R, I10T, V17I, I20V, H44Y, A46P, T76A, K78Q, A81T, V99I, H100Y, H155Y, T188I, M258I, T289M, G336S, V338M, P340S, N366S, G382E, S405T, I418M, S434N, D460G | 2.3.4.4b | United Kingdom / England | Byrne, Alex |
| A/swan/Poland/MB078_22VIR2515-7/2022 | EPI_ISL_11922813 | 2022-02-10 | *Cygnus olor* | K6R, I10T, V17I, I20V, H44Y, A46P, T76A, K78Q, A81T, V99I, H100Y, H155Y, T188I, M258I, T289M, G336S, V338M, P340S, N366S, G382E, S405T, I418M, S434N, D460G | 2.3.4.4b | Poland | Swieton, E*. et al.* |
| A/seagull/Italy/21VIR11259-10/2021 | EPI_ISL_8882213 | 2021-12-15 | Seagull | K6R, I10T, V17I, I20V, H44Y, A46P, T76A, K78Q, A81T, V99I, H100Y, N141S, H155Y, T188I, M258I, T289M, G336S, V338M, P340S, N366S, G382E, S405T, I418M, S434N, D460G | 2.3.4.4b | Italy | Barbierato, G.*et al.* |
| A/Withe-tiled_eagle/Estonia/TA2124126-1_21VIR10433-11/2021 | EPI_ISL_7778754 | 2021-10-12 | Withe-tiled eagle | K6R, I10T, V17I, I20V, H44Y, A46P, T76A, K78Q, A81T, V99I, H100Y, H155Y, T188I, M258I, T289M, G336S, V338M, P340S, N366S, G382E, S405T, I418M, S434N, D460G | 2.3.4.4b | Estonia | Nurmoja, I. *et al.* |
| A/greylag goose/Netherlands/21037809-001/2021 | EPI_ISL_6761000 | 2021-10-31 | *Anser anser* | K6R, I10T, V17I, I20V, H44Y, A46P, T76A, K78Q, A81T, V99I, H100Y, H155Y, T188I, M258I, T289M, G336S, V338M, P340S, N366S, G382E, S405T, I418M, S434N, D460G | 2.3.4.4b | Netherlands / South Holland | Beerens, Nancy *et al.* |
| A/mute swan/Croatia/100/2021 | EPI_ISL_6507374 | 2021-11-12 | *Cygnus olor* | K6R, I10T, V17I, I20V, H44Y, A46P, T76A, K78Q, A81T, V99I, H100Y, H155Y, T188I, M258I, T289M, G336S, V338M, P340S, N355K, G356W, V357G, N366S, G382E, S405T, I418M, S434N, D460G | 2.3.4.4b | Croatia / Sisacko-Moslavacka Zupanija / Muzilovcica | Savić, Vladimir |
| A/mute_swan/Poland/MB550/2021 | EPI_ISL_18245814 | 2021-12-23 | *Cygnus olor* | K6R, I10T, V17I, I20V, H44Y, A46P, T76A, K78Q, A81T, V99I, H100Y, H155Y, T188I, M258I, T289M, G336S, V338M, P340S, N366S, G382E, S405T, I418M, S434N, D460G | 2.3.4.4b | Poland / West Pomeranian Voivodeship | Swieton, E*. et al.* |
| A/pelican/Montenegro/833/2022 | EPI_ISL_17731667 | 2022-04-04 | Pelican | K6R, I10T, V17I, I20V, H44Y, A46P, T76A, K78Q, A81T, V99I, H100Y, H155Y, T188I, M258I, T289M, G336S, V338M, P340S, N366S, G382E, S405T, I418M, S434N, D460G | 2.3.4.4b | Montenegro / Skadar lake | Bojan, Adžić *et al.* |
| A/LutraLutra/England/4197OP/2022 | EPI_ISL_17637991 | 2022-01-24 | *Lutra lutra* | K6R, I10T, V17I, I20V, H44Y, A46P, T76A, K78Q, A81T, V99I, H100Y, H155Y, T188I, M258I, T289M, G336S, V338M, P340S, N366S, G382E, S405T, I418M, S434N, D460G | 2.3.4.4b | United Kingdom / Cumbria | Catherine, Moore *et al.* |
| A/goose/Chelyabinsk/1341-3/2021 | EPI_ISL_16838578 | 2021-08-08 | Goose | K6R, I10T, V17I, I20V, H44Y, A46P, T76A, K78Q, A81T, V99I, H100Y, H155Y, T188I, M258I, T289M, G336S, V338M, P340S, N366S, G382E, S405T, I418M, S434N, D460G | 2.3.4.4b | Russian Federation / Chelyabinsk Oblast | Zinyakov, N. *et al.* |
| A/wigeon/Sakhalin/37M/2021 | EPI_ISL_15081424 | 2021-09-17 | Wigeon | K6R, I10T, V17I, I20V, H44Y, A46P, T76A, K78Q, A81T, V99I, H100Y, H155Y, T188I, M258I, T289M, N309S, G336S, V338M, P340S, N366S, G382E, S405T, I418M, S434N, D460G | 2.3.4.4b | Russian Federation | Sobolev, Ivan |
| A/turkey/Italy/IZSLT_22VIR366-3/2022 | EPI_ISL_14761272 | 2022-01-04 | Turkey | K6R, T9I, I10T, V17I, I20V, H44Y, A46P, T76A, K78Q, A81T, V99I, H100Y, H155Y, T188I, M258I, T289M, G336S, V338M, P340S, N366S, G382E, S405T, I418M, S434N, D460G | 2.3.4.4b | Italy | Barbierato, G.*et al* |
| A/laying_hen/Italy/21VIR11498-1/2021 | EPI_ISL_14760946 | 2021-12-21 | Laying hen | K6R, I10T, V17I, I20V, H44Y, A46P, T76A, K78Q, A81T, V99I, H100Y, K111N, H155Y, T188I, M258I, T289M, G336S, V338M, P340S, N366S, G382E, S405T, I418M, S434N, D460G | 2.3.4.4b | Italy | Barbierato, G.*et al* |
| A/goose/Italy/IZSLT_21VIR10273/2021 | EPI_ISL_14760611 | 2021-11-22 | Goose | K6R, I10T, V17I, I20V, H44Y, A46P, T76A, K78Q, A81T, V99I, H100Y, K111N, H155Y, T188I, M258I, T289M, G336S, V338M, P340S, N366S, G382E, S405T, I418M, S434N, D460G | 2.3.4.4b | Italy | Barbierato, G.*et al* |
| A/buzzard/England/107870/2021 | EPI_ISL_13369398 | 2021-11-02 | *Buteo buteo* | K6R, I10T, V17I, I20V, H44Y, A46P, T76A, K78Q, A81T, V99I, H100Y, H155Y, T188I, M258I, T289M, G336S, V338M, P340S, N366S, G382E, S405T, I418M, S434N, D460G | 2.3.4.4b | United Kingdom / England | Byrne, Alex |
| A/turkey/Israel/537/2021 | EPI_ISL_12749687 | 2021-10-12 | *Meleagris gallopavo* | K6R, I10T, V17I, I20V, H44Y, A46P, I54V, T76A, K78Q, A81T, V99I, H100Y, H155Y, T188I, M258I, T289M, G336S, V338M, P340S, N366S, G382E, S405T, I418M, S434N, D460G | 2.3.4.4b | Israel / Northern District | Zuckerman, N.S. *et al.* |
| A/chicken/Czech_Republic/3306-2/2022 | EPI_ISL_12325210 | 2022-02-09 | Chicken | K6R, I10T, V17I, I20V, H44Y, A46P, T76A, K78Q, A81T, V99I, H100Y, H155Y, T188I, M258I, T289M, G336S, V338M, P340S, N366S, G382E, S405T, I418M, S434N, D460G | 2.3.4.4b | Czech Republic / Stredocesky Kraj / Okres Kolin | Nagy, Alexander *et al.* |
| A/duck/Czech_Republic/3306-1/2022 | EPI_ISL_12324302 | 2022-02-09 | Duck | K6R, I10T, V17I, I20V, H44Y, A46P, T76A, K78Q, A81T, V99I, H100Y, H155Y, T188I, M258I, T289M, G336S, V338M, P340S, N366S, G382E, S405T, I418M, S434N, D460G | 2.3.4.4b | Czech Republic / Stredocesky Kraj / Okres Kolin | Nagy, Alexander *et al.* |
| A/grey_heron/Czech_Republic/25338-2/2021 | EPI_ISL_12223734 | 2021-12-18 | Grey heron | K6R, I10T, V17I, I20V, H44Y, A46P, T76A, K78Q, A81T, V99I, H100Y, H155Y, T188I, M258I, T289M, G336S, V338M, P340S, N366S, G382E, S405T, I418M, S434N, D460G | 2.3.4.4b | Czech Republic / Liberecky Kraj / Okres Ceska Lipa | Nagy, Alexander *et al.* |
| A/grey_heron/Czech_Republic/25338-1/2021 | EPI_ISL_12223688 | 2021-12-18 | Grey heron | K6R, I10T, V17I, I20V, H44Y, A46P, T76A, K78Q, A81T, V99I, H100Y, H155Y, T188I, M258I, T289M, G336S, V338M, P340S, N366S, G382E, S405T, I418M, S434N, D460G | 2.3.4.4b | Czech Republic / Liberecky Kraj / Okres Ceska Lipa | Nagy, Alexander *et al.* |
| A/gray_heron/Denmark/24326-1.02/2021-10-28 | EPI_ISL_11798577 | 2021-10-28 | Grey heron | K6R, I10T, V17I, I20V, H44Y, A46P, T76A, K78Q, A81T, V99I, H100Y, H155Y, T188I, M258I, T289M, G336S, V338M, P340S, N366S, G382E, S405T, I418M, S434N, D460G | 2.3.4.4b | Denmark / Region Midtjylland / Hostebro Kommune | Charlotte Hjulsager and Yuan Liang |
| A/red_fox/Netherlands/21040099-007/2021 | EPI_ISL_7683138 | 2021-12-03 | *Vulpes vulpes* | K6R, I10T, V17I, I20V, H44Y, A46P, T76A, K78Q, A81T, V99I, H100Y, H155Y, T188I, M258I, T289M, G336S, V338M, P340S, N366S, G382E, S405T, I418M, S434N, D460G | 2.3.4.4b | Netherlands / North Brabant | Beerens, Nancy *et al.* |
| A/red_fox/Netherlands/21040099-006/2021 | EPI_ISL_7683137 | 2021-12-03 | *Vulpes vulpes* | K6R, I10T, V17I, I20V, H44Y, A46P, T76A, K78Q, A81T, V99I, H100Y, H155Y, T188I, M258I, T289M, G336S, V338M, P340S, N366S, G382E, S405T, I418M, S434N, D460G | 2.3.4.4b | Netherlands / North Brabant | Beerens, Nancy *et al.* |
| A/turkey/Italy/21VIR11591-8/2021 | EPI_ISL_8882171 | 2021-12-23 | Turkey | K6R, I10T, V17I, I20V, H44Y, A46P, T76A, K78Q, A81T, V99I, H100Y, K111N, H155Y, T188I, M258I, T289M, G336S, V338M, P340S, N366S, G382E, S405T, I418M, S434N, D460G | 2.3.4.4b | Italy | Barbierato, G. *et al.* |
| A/great_egret/Czech_Republic/23609/2021 | EPI_ISL_8515483 | 2021-11-28 | Great egret | K6R, I10T, V17I, I20V, H44Y, A46P, T76A, K78Q, A81T, V99I, H100Y, H155Y, T188I, M258I, T289M, G336S, V338M, P340S, N366S, G382E, S405T, I418M, S434N, D460G | 2.3.4.4b | Czech Republic / Liberecky Kraj / Okres Ceska Lipa | Nagy, Alexander *et al.* |
| A/mute_swan/Netherlands/21039841-002/2021 | EPI_ISL_7952124 | 2021-11-30 | *Cygnus olor* | K6R, I10T, V17I, I20V, H44Y, A46P, T76A, K78Q, A81T, V99I, H100Y, H155Y, T188I, M258I, T289M, G336S, V338M, P340S, N366S, G382E, D398N, S405T, I418M, S434N, D460G | 2.3.4.4b | Netherlands / Provincie Utrecht | Beerens, Nancy *et al.* |
| A/barnacle_goose/Germany-SH/AI06005/2021 | EPI_ISL_7753251 | 2021-10-19 | Barnacle goose | K6R, I10T, V17I, I20V, H44Y, A46P, T76A, K78Q, A81T, V99I, H100Y, H155Y, T188I, M258I, T289M, G336S, V338M, P340S, N366S, G382E, S405T, I418M, S434N, D460G | 2.3.4.4b | Germany / Schleswig-Holstein | King, Jacqueline |
| A/goose/Italy/IZSLT-21VIR10273/2021 | EPI_ISL_7733645 | 2021-11-22 | Goose | K6R, I10T, V17I, I20V, H44Y, A46P, T76A, K78Q, A81T, V99I, H100Y, K143R, H155Y, T188I, M258I, T289M, G336S, V338M, P340S, N366S, G382E, S405T, I418M, S434N, D460G | 2.3.4.4b | Italy / Rome | Zecchin, B. *et al.* |
| A/red_fox/Netherlands/21040099-001/2021 | EPI_ISL_7683118 | 2021-12-03 | *Vulpes vulpes* | K6R, I10T, V17I, I20V, H44Y, A46P, T76A, K78Q, A81T, V99I, H100Y, H155Y, T188I, M258I, T289M, G336S, V338M, P340S, N366S, G382E, S405T, I418M, S434N, D460G | 2.3.4.4b | Netherlands / North Brabant | Beerens, Nancy *et al.* |
| A/goose/Netherlands/21039029-001/2021 | EPI_ISL_7267252 | 2021-11-17 | Goose | K6R, I10T, V17I, I20V, H44Y, A46P, T76A, K78Q, A81T, V99I, H100Y, H155Y, T188I, M258I, T289M, G336S, V338M, P340S, N366S, G382E, S405T, I418M, S434N, D460G | 2.3.4.4b | Netherlands / Provincie Noord-Holland | Beerens, Nancy *et al.* |
| A/greylag_goose_/Sweden/SVA211118SZ0354/FB004497/I-2021 | EPI_ISL_7055384 | 2021-11-17 | Greylag goose | K6R, I10T, V17I, I20V, H44Y, A46P, T76A, K78Q, A81T, V99I, H100Y, H155Y, T188I, S247N, M258I, T289M, G336S, V338M, P340S, N366S, G382E, S405T, I418M, S434N, D460G | 2.3.4.4b | Sweden / Kalmar Lan / Kalmar Kommun | Zohari, Siamak |
| A/bean_goose/Sweden/SVA211111SZ0372/FB004482/2021 | EPI_ISL_7049600 | 2021-11-05 | Greylag goose | K6R, I10T, V17I, I20V, H44Y, A46P, T76A, K78Q, A81T, V99I, H100Y, H155Y, T188I, M258I, T289M, G336S, V338M, P340S, N366S, G382E, S405T, I418M, S434N, D460G | 2.3.4.4b | Sweden / Skane Lan / Simrishamns Kommun | Zohari, Siamak |
| A/mute_swan/Netherlands/21038706-002/2021 | EPI_ISL_6761019 | 2021-11-14 | *Cygnus olor* | K6R, I10T, V17I, I20V, H44Y, A46P, T76A, K78Q, A81T, V99I, H100Y, H155Y, T188I, M258I, T289M, G336S, V338M, P340S, N366S, G382E, S405T, I418M, S434N, D460G | 2.3.4.4b | Netherlands / South Holland | Beerens, Nancy *et al.* |
| A/goose/Tyumen/33-52V/2021 | EPI_ISL_5463805 | 2021-10-07 | *Anser anser domesticus* | K6R, I10T, V17I, I20V, H44Y, A46P, T76A, K78Q, A81T, V99I, H100Y, H155Y, T188I, M258I, T289M, G336S, V338M, P340S, N366S, G382E, S405T, I418M, S434N, D460G | 2.3.4.4b | Russian Federation / Tyumen Oblast | Natalia, Goncharova *et al.* |
| A/grey_heron/Czech_Republic/23608/2021 | EPI_ISL_8515481 | 2021-11-28 | Grey heron | K6R, I10T, V17I, I20V, H44Y, A46P, T76A, K78Q, A81T, V99I, H100Y, H155Y, T188I, M258I, T289M, G336S, V338M, P340S, N366S, G382E, S405T, I418M, S434N, D460G | 2.3.4.4b | Czech Republic / Liberecky Kraj / Okres Ceska Lipa | Nagy, Alexander *et al.* |
| A/turkey/Germany-MV/AI06035/2021 | EPI_ISL_7753290 | 2021-10-20 | Turkey | K6R, I10T, V17I, I20V, H44Y, A46P, T76A, K78Q, A81T, V99I, H100Y, H155Y, T188I, M258I, T289M, G336S, V338M, P340S, N366S, G382E, S405T, I418M, S434N, D460G | 2.3.4.4b | Germany / Mecklenburg-Vorpommern | King, Jacqueline |
| A/chicken/Italy/IZSLT-122448_21VIR9218-1/2021 | EPI_ISL_7733644 | 2021-10-28 | Chicken | K6R, I10T, V17I, I20V, H44Y, A46P, T76A, K78Q, A81T, T84I, V99I, H100Y, H155Y, T188I, M258I, T289M, G336S, V338M, P340S, N366S, G382E, S405T, I418M, S434N, D460G | 2.3.4.4b | Italy / Rome | Zecchin, B. *et al.* |
| A/common_buzzard/Denmark/08287-1.01/2022 | EPI_ISL_16115054 | 2022-08-30 | *Buteo buteo* | K6R, I10T, V17I, I20V, H44Y, A46P, T76A, K78Q, A81T, V99I, H100Y, H155Y, T188I, M258I, T289M, G336S, V338M, P340S, N366S, G382E, S405T, I418M, S434N, D460G | 2.3.4.4b | Denmark / Region Syddanmark / Sonderborg Kommune | Hjulsager, Charlotte and  Liang, Yuan |
| A/domestic_goose/Poland/H371/2022 | EPI_ISL_14917951 | 2022-07-21 | *Anser anser domesticus* | K6R, I10T, V17I, I20V, H44Y, A46P, T76A, K78Q, A81T, V99I, H100Y, V149I, H155Y, T188I, M258I, T289M, G336S, V338M, P340S, N366S, G382E, S405T, I418M, S434N, D460G | 2.3.4.4b | Poland / Greater Poland Voivodeship | Swieton E., Smietanka K. |
| A/breeding_hen/Poland/H364/2022 | EPI_ISL_14917942 | 2022-07-21 | *Gallus gallus domesticus* | K6R, I10T, V17I, I20V, H44Y, A46P, T76A, K78Q, A81T, V99I, H100Y, V149I, H155Y, T188I, M258I, E287V, T289M, G336S, V338M, P340S, N366S, G382E, S405T, I418M, S434N, D460G | 2.3.4.4b | Poland / Greater Poland Voivodeship | Swieton E., Smietanka K. |
| A/breeding_hen/Poland/H361/2022 | EPI_ISL_14917915 | 2022-07-18 | *Gallus gallus domesticus* | K6R, I10T, V17I, I20V, H44Y, A46P, T76A, K78Q, A81T, V99I, H100Y, V149I, H155Y, T188I, M258I, E287V, T289M, G336S, V338M, P340S, N366S, G382E, S405T, I418M, S434N, D460G | 2.3.4.4b | Poland / Greater Poland Voivodeship | Swieton E., Smietanka K. |
| A/buzzard/Italy/22VIR767-1/2022 | EPI_ISL_14761361 | 2022-01-24 | Buzzard | K6R, I10T, I20V, H44Y, A46P, T76A, K78Q, A81T, V99I, H100Y, K111N, H155Y, T188I, M258I, T289M, G336S, V338M, P340S, N366S, G382E, S405T, I418M, S434N, D460G | 2.3.4.4b | Italy | Barbierato, G. *et al.* |
| A/duck/Israel/589/2021 | EPI_ISL_13618384 | 2021-11-18 | Duck | K6R, I10T, V17I, I20V, H44Y, A46P, T76A, K78Q, A81T, V99I, H100Y, H155Y, T188I, M258I, T289M, T332P, G336S, V338M, P340S, N366S, G382E, S405T, I418M, S434N, D460G | 2.3.4.4b | Israel / Northern District | Shkoda, I. *et al.* |
| A/chicken/England/069816/2021 | EPI_ISL_13370571 | 2021-12-12 | *Gallus gallus domesticus* | K6R, I10T, V17I, I20V, H44Y, A46P, T76A, K78Q, A81T, V99I, H100Y, H155Y, T188I, M258I, T289M, G336S, V338M, P340S, N366S, G382E, S405T, I418M, S434N, D460G | 2.3.4.4b | United Kingdom / England | Byrne, Alex |
| A/turkey/England/068583/2021 | EPI_ISL_13370559 | 2021-12-09 | *Meleagris gallopavo* | K6R, I10T, V17I, I20V, H44Y, A46P, T76A, K78Q, A81T, V99I, H100Y, H155Y, T188I, M258I, T289M, G336S, V338M, P340S, N366S, G382E, S405T, I418M, S434N, D460G | 2.3.4.4b | United Kingdom / England | Byrne, Alex |
| A/buzzard/Wales/055388/2021 | EPI_ISL_13370343 | 2021-11-29 | *Buteo buteo* | K6R, I10T, V17I, I20V, H44Y, A46P, T76A, K78Q, A81T, V99I, H100Y, H155Y, T188I, M258I, N270D, T289M, G336S, V338M, P340S, N366S, G382E, S405T, I418M, S434N, D460G | 2.3.4.4b | United Kingdom / Wales | Byrne, Alex |
| A/chicken/England/062137/2021 | EPI_ISL_13369777 | 2021-11-25 | *Gallus gallus domesticus* | K6R, I10T, V17I, I20V, H44Y, A46P, T76A, K78Q, A81T, V99I, H100Y, H155Y, T188I, M258I, T289M, G336S, V338M, P340S, N366S, G382E, S405T, I418M, S434N, D460G | 2.3.4.4b | United Kingdom / England | Byrne, Alex |
| A/mute_swan/England/244029/2021 | EPI_ISL_13369756 | 2021-11-14 | *Cygnus olor* | K6R, I10T, V17I, I20V, H44Y, A46P, T76A, K78Q, A81T, V99I, H100Y, H155Y, T188I, M258I, N270D, T289M, G336S, V338M, P340S, N366S, G382E, S405T, I418M, S434N, D460G | 2.3.4.4b | United Kingdom / England | Byrne, Alex |
| A/Herring_gull/Wales/054692/2021 | EPI_ISL_13369400 | 2021-11-02 | *Larus argentatus* | K6R, I10T, V17I, I20V, H44Y, A46P, T76A, K78Q, A81T, V99I, H100Y, H155Y, T188I, M258I, T289M, G336S, V338M, P340S, N366S, G382E, S405T, I418M, S434N, D460G | 2.3.4.4b | United Kingdom / Wales | Byrne, Alex |
| A/chicken/Netherlands/21038165-006010/2021 | EPI_ISL_13360259 | 2021-11-07 | *Gallus gallus* | K6R, I10T, V17I, I20V, V34A, H44Y, A46P, T76A, K78Q, A81T, V99I, H100Y, H155Y, T188I, M258I, T289M, G336S, V338M, P340S, N366S, G382E, S405T, I418M, S434N, D460G | 2.3.4.4b | Netherlands / Provincie Groningen | Beerens, Nancy *et al.* |
